# Fragments of Self: A Case Report of Episodic Autobiographical Memory Disturbance

**DOI:** 10.1192/bjo.2026.11774

**Published:** 2026-06-30

**Authors:** Sreya Salim, Craig Dickson, Ian Collings

**Affiliations:** 1 Powys Teaching Health Board, Ystradgynlais, United Kingdom; 2 Health Education and Improvement Wales, Cardiff, United Kingdom

## Abstract

**Aims:**

Increasing evidence suggests that autobiographical memory exists on a continuum rather than a dichotomy of normal and abnormal. We present a case of lifelong deficiency of episodic autobiographical memory.

**Methods:**

A 60-year-old military veteran was referred to a memory clinic with a lifelong difficulty in recalling personal life events across his lifespan, including childhood, military service, and later life. The lack of recall was more marked for remote memories. This concern came to attention following recent interpersonal conflicts, which triggered anxiety about possible dementia. Knowledge of his past was largely derived from factual information obtained from family and friends.

Cognitive assessment using the Addenbrooke’s Cognitive Examination yielded a score of 95/100. CT brain imaging revealed a foreign body, likely an air-gun pellet, beneath the right orbital socket, with no recollection of the associated incident. There were no functional impairments, behavioural changes, or affective symptoms. An autobiographical interview spanning multiple life stages demonstrated reliance on external semantic details to compensate for episodic recall. Qualitative indices of episodic richness, vividness, emotional salience, and temporal integration were markedly reduced, particularly for remote autobiographical memories.

Following discussion of possible supportive options, including psychological interventions, further support was declined. He expressed a preference for understanding his condition rather than altering his way of life.

**Results:**

This case illustrates a lifelong deficit in episodic autobiographical memory with preserved semantic memory and intact general cognition. Dementia was ruled out due to the lack of marked impairment in cognitive domains. The amnesia was selective for autobiographical episodic memory and extended across all life stages, making dissociative amnesia unlikely. There was no evidence of intrusive memories, avoidance, hyperarousal, or trauma-related symptomatology to suggest PTSD. A diagnosis of severely deficient autobiographical memory (SDAM) was therefore made.

SDAM refers to an inability to mentally re-experience personal events despite intact semantic learning and recall. First described in 2015, only a small number of cases have been reported to date, although it is presumed to be under-recognised in clinical practice. This case is notable for the diagnostic uncertainty it posed and for highlighting patient perspectives on engagement with treatment.

**Conclusion:**

Severely deficient autobiographical memory illustrates the functional spectrum of autobiographical memory and challenges traditional categorical models. This case underscores interindividual differences in remembering the past, highlights the risk of misdiagnosis, and emphasises the need for greater clinical awareness and research into spectrum-based conceptualisations of autobiographical memory.

